# Supplementary figures and images for: Mechanical effects of MitraClip on leaflet stress and myocardial strain in functional mitral regurgitation – A finite element modeling study
Source: PLoS One. 2019 Oct 10;14(10):e0223472. doi: 10.1371/journal.pone.0223472 (PMC6786765; doi:10.1371/journal.pone.0223472)

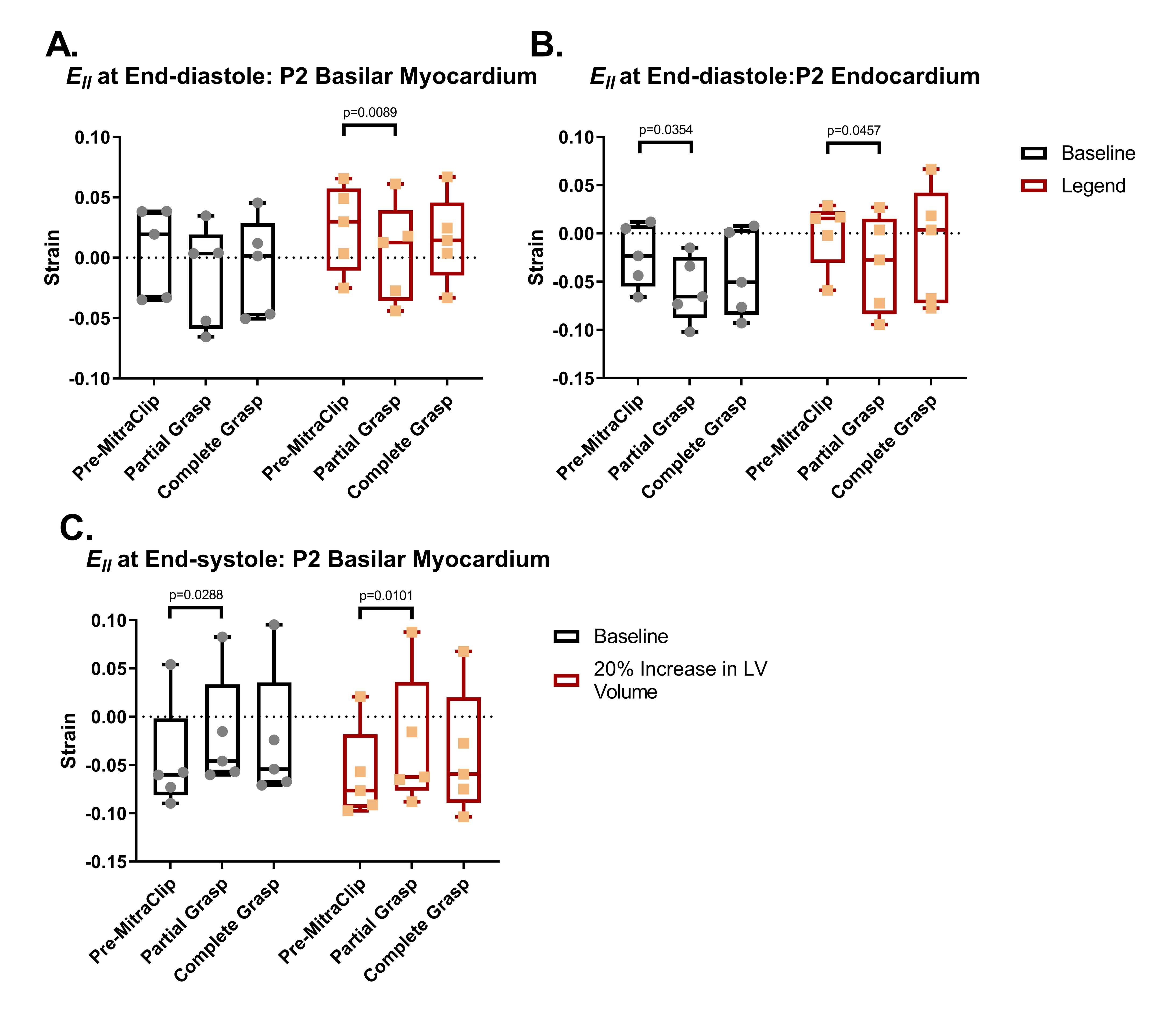

Supplement: S1 Fig — Longitudinal strain, E_ll, after simulated partial and complete grasp MitraClip in the P2 sub-valvular myocardium at end-diastole (A), P2 sub-valvular endocardium at end-diastole (B) and P2 sub-valvular myocardium end-systole (C) respectively. Ell before and after MitraClip is relative to the pre-procedure unloaded state. (TIF) [file pone.0223472.s001.tif]
